# Early Appearance of Nonvisual and Circadian Markers in the Developing Inner Retinal Cells of Chicken

**DOI:** 10.1155/2014/646847

**Published:** 2014-05-20

**Authors:** Nicolás M. Díaz, Luis P. Morera, Daniela M. Verra, María A. Contin, Mario E. Guido

**Affiliations:** CIQUIBIC (CONICET), Departamento de Química Biológica, Facultad de Ciencias Químicas, Universidad Nacional de Córdoba, Ciudad Universitaria, 5000 Córdoba, Argentina

## Abstract

The retina is a key component of the vertebrate circadian system; it is responsible for detecting and transmitting the environmental illumination conditions (day/night cycles) to the brain that synchronize the circadian clock located in the suprachiasmatic nucleus (SCN). For this, retinal ganglion cells (RGCs) project to the SCN and other nonvisual areas. In the chicken, intrinsically photosensitive RGCs (ipRGCs) expressing the photopigment melanopsin (Opn4) transmit photic information and regulate diverse nonvisual tasks. In nonmammalian vertebrates, two genes encode *Opn4*: the *Xenopus* (*Opn4x*) and the mammalian (*Opn4m*) orthologs. RGCs express both *Opn4* genes but are not the only inner retinal cells expressing *Opn4x*: horizontal cells (HCs) also do so. Here, we further characterize primary cultures of both populations of inner retinal cells (RGCs and HCs) expressing *Opn4x*. The expression of this nonvisual photopigment, as well as that for different circadian markers such as the clock genes *Bmal1*, *Clock*, *Per2*, and *Cry1*, and the key melatonin synthesizing enzyme, arylalkylamine *N*-acetyltransferase (AA-NAT), appears very early in development in both cell populations. The results clearly suggest that nonvisual Opn4 photoreceptors and endogenous clocks converge all together in these inner retinal cells at early developmental stages.

## 1. Introduction

The circadian system of vertebrates that controls most physiological and behavioral rhythms includes the retina, the pineal gland, and the hypothalamic suprachiasmatic nucleus (SCN) together with a number of peripheral oscillators distributed throughout the body [1, 2]. Light is the main synchronizer of the circadian system while the retina is responsible for sensing the environmental lighting conditions which change along the day/night cycles, by more than six orders of magnitude, to adjust endogenous clocks located in the brain. The retina contains the autonomous clock machinery that generates a variety of self-sustained biochemical and cellular rhythms, allowing it to measure time and predict the 24 h changes in the ambient light conditions [1, 3, 4]. Embryonic retinal cells maintained in culture and different retinal cell populations display robust circadian rhythms in a number of molecular and biochemical aspects and express circadian-based clock genes [5–10]. In this respect, photoreceptor cells (PRCs) synthesize melatonin almost exclusively in the retina of different vertebrate species with the highest levels at night [1, 8, 11–13]; by contrast, retinal ganglion cells (RGCs) in the chicken synthesize small amounts of melatonin rhythmically with higher levels during the day, in clear antiphase to the nocturnal PRC profiles [1, 8, 11–13].

Briefly, the molecular clock that also operates in the vertebrate retina consists of a transcriptional/translational feedback circuitry that generates circadian patterns of gene expression by means of the action of positive elements such as Clock, Bmal1, and NPAS2. These latter interact with the negative elements Periods (Per) 1 and 2 and cryptochrome (Cry) 1 and 2, in which the casein kinases CKI and CKI*δ* set the circadian period by phosphorylating PER proteins to regulate their degradation and nuclear localization [1, 14].

In the last decades, two major discoveries have had an extraordinary impact in the field of vision and chronobiology: the identification of a novel photopigment named melanopsin (Opn4) which is only expressed in a subpopulation of RGCs in mammals [15] and the demonstration that these Opn4 (+) RGCs were intrinsically photosensitive cells (ipRGCs) [16]. Working with nonmammalian vertebrates, we were the first to demonstrate that a subpopulation of RGCs in the chicken retina was ipRGCs acting through a photocascade similar to that of rhabdomeric photoreceptors of invertebrates involving the activation of phospholipase C and Ca^2+^ mobilization [17, 18]. Moreover, we and other laboratories have shown that the two genes for Opn4, the Xenopus (*Opn4x*) and the mammalian (*Opn4m*) orthologs, are expressed in chicken retina at the level of mRNA [5, 6, 17, 19, 20] and protein [7, 21]. Moreover, the expression of Opn4 proteins was reported to vary during development [21]. In fact, Opn4m was shown to be restricted exclusively to the ganglion cell layer (GCL) all through development, whereas Opn4x was limited to the formation of GCL and optic nerve at early embryonic days (E8), though by E15 its expression was mostly in Prox1 (+) horizontal cells (HCs) [21]. Concomitantly with HC birth and migration between E10–15, Opn4x (+) immunoreactivity appeared in the cell somas of Prox1 (+) HC and displayed prominent labelling of the lower outer plexiform layer (OPL). Indeed, these Opn4x cells resembled typical HCs: morphologically, some are axonless candelabrum-shaped HCs shown to mainly connect to cone pedicles [22, 23]. Based on a number of specification markers, horizontal and amacrine cells in the inner retina could be considered sister cells of ipRGCs derived from a common ancestor photoreceptor progenitor [24].

In this work, we further characterize primary cultures of RGCs and HCs obtained from embryonic retinas at early stages in development by different procedures of purification; strikingly, both retinal cell populations express the nonvisual photopigment Opn4 (*Opn4m* and/or* Opn4x*) as well as components of the clock machinery needed to measure time to temporally adjust retinal physiology. To this end, the aim of this work was to investigate whether nonvisual Opn4 photoreceptors and endogenous clocks converge in specific cell populations of the chicken inner retina.

## 2. Materials and Methods

### 2.1. Materials

All reagents were of analytical grade. The secondary antibodies used for immunocytochemistry (ICC) and immunohistochemistry (IHC) were Alexa Fluor 488 goat anti-rabbit and Alexa Fluor 546 goat anti-mouse IgG (dilution 1 : 1000; Invitrogen-Molecular Probes, Eugene, OR, USA), Prox-1 Polyclonal Antibody, anti-rabbit (dilution 1/2500, Millipore, Temecula, CA, USA) NeuN monoclonal antibody, mouse (1/100, Millipore, Temecula, CA); glutamine synthetase (GS) monoclonal antibody, mouse (1/500, Millipore, Temecula, CA, USA); GABA polyclonal antibody, rabbit (1/500, Abcam Cambridge MA, USA); anti-mouse Cry1 cat CRY11-A (1/100 Alpha Diagnostic Intl. inc), anti-mouse Per2 cat PER21-A (1/100 Alpha Diagnostic Intl. Inc.), and anti-BMAL1 AB2298 (1/100 Millipore Temecula California). *α*-Tubulin (*α*-Tub) was detected by the mouse monoclonal DM1A antibody (Sigma Aldrich, 1 : 1000 for WB). The primary antibody against chicken* Opn4x* was raised in rabbit using the specific* Opn4x* peptide 1: RQKRDLLPDSYSCSEE [21]. The antibody against the chicken* Opn4m* was raised in rat and generated with the specific* Opn4m* peptide: CKHGNRELQKQYHR (Bio-Synthesis Inc., Lewisville, TX, USA) [21]. Preparation of anti-chicken Thy-1 sera was performed by Bio-Synthesis, Inc. (Bio-Synthesis Inc., Lewisville, TX, USA), by using the NH2-KNITVIKDKLEKC-OH peptide sequence conjugated with KLH [17].

Propidium iodide (PI), DAPI, protease inhibitor, papain suspension in 0.05 M sodium acetate, and laminin were from Sigma Aldrich (St. Louis, MO). Aqueous mounting medium (FluorSave) was from Calbiochem (San Diego, CA). B-27 supplement 50 was from Invitrogen-Gibco (Grand Island, NY), Leibovitz's (L-15) from Life Technologies, Invitrogen GIBCO (Carlsbad, CA).

### 2.2. Animal Handling

For studies involving immunochemistry, we used 10-day-old chickens (*Gallus gallus domesticus*). Chickens were anesthetized with 2.5 mL/Kg Equitesin (426 mg chloral hydrate, 96 mg pentobarbital, 212 mg MgSO_4_, 3.5 mL propylene glycol, and 1 mL ethanol, final volume 10 mL) and sacrificed by decapitation.

All experiments were performed in accordance with the Use of Animals in Ophthalmic and Vision Research of ARVO, approved by the local animal care committee (School of Chemistry, Universidad Nacional de Córdoba; Exp. 15-99-39796).

### 2.3. Primary Cultures of Embryonic RGCs

RGCs were purified from embryonic day 8 (E8); neural chicken retinas were dissected in ice-cold Ca^+2^- Mg^+2^ free Tyrode's buffer containing 25 mM glucose as previously reported [9, 25]. Briefly, cells were trypsin-treated and rinsed with soybean trypsin inhibitor and Dulbecco's modified Eagle's medium (DMEM). After dissociation, the cell suspension from 30 to 60 retinas was poured into petri dishes pretreated with 2.5 *μ*g/mL protein A followed by incubation at 37°C for 30 min with an anti-chicken Thy-1 polyclonal antibody. After being washed exhaustively, identical aliquots of the remaining bound RGCs were harvested in DMEM containing B27 (Life Technologies, Invitrogen, GIBCO, Carlsbad, CA; dilution: 1/500 v/v) and seeded in petri dishes previously treated with 10 *μ*g/mL polylysine and 5 *μ*g/mL laminin. The RGC cultures were incubated at 37°C under constant 5% CO_2_-air flow in a humid atmosphere [17, 18, 21]. In an alternative procedure, the cell suspension from 20 to 40 retinas was poured into petri dishes pretreated with 2.5 *μ*g/mL protein A followed by incubation at 37°C for 30 min with an anti-chicken* Opn4x* polyclonal antibody (Bio-Synthesis Inc., Lewisville, TX, USA). After exhaustive washing, similar aliquots of the remaining bound cells were harvested in DMEM containing B27 (Life Technologies, Invitrogen, GIBCO, Carlsbad, CA; dilution: 1/50 v/v), forskolin from* Coleus forskohlii* (Sigma Aldrich, St. Louis, MO, 4.25 *μ*g/mL in DMSO), and Recombinant Human BDNF (R&D Systems, Minneapolis, MN, 50 *μ*g/mL) and seeded in petri dishes previously treated with 10 *μ*g/mL polylysine and 5 *μ*g/mL laminin. Primary cell cultures were incubated at 37°C under constant 5% CO_2_-air flow in a humid atmosphere for 3 days and further characterized with specific retinal cell type markers by immunochemistry.

### 2.4. Primary Cultures of Embryonic Horizontal Cells (HCs)

HCs were purified from the chicken neural retinas at embryonic day 15 (E15) as previously reported [26]. Briefly, eyes were dissected out from the head and sectioned in ice-cold Ca^+2^- Mg^+2^ free Hank's buffered saline solution containing 25 mM glucose (CMF-HBSS) at the level of the ora serrata, the vitreous body was removed, and the retina was peeled from the eyecup by gentle shaking in order to avoid detachment of the pigment epithelium. The retina was cut into 6–8 pieces and incubated with CMF containing 3 U/mL of papain for 25 min at 37°C and then kept on ice until use.

In order to isolate HCs, cells were dissociated from the retinal tissue by a mechanical triturating procedure with a fire-polished Pasteur pipette. After dissociation, the cell suspension was subjected to a bovine serum albumin (BSA) discontinuous gradient of concentrations ranging from 1 to 4%. After dissociation, cells were centrifuged at 300 rpm for 15 min and different phases were collected and cultured for 4 days in L15 containing B27 (Life Technologies, Invitrogen, GIBCO, Carlsbad, CA; dilution: 1/500 v/v) in order to allow neurite outgrowth and morphological differentiation. Cultures were incubated over 4 days at 37°C in a humid atmosphere containing 5% CO_2_. Characterization of harvested cells was performed by immunostaining with different HC and other retinal cell type markers. The cell cultures were highly enriched in HCs (≥75%) as previously shown [26].

### 2.5. Immunocytochemistry (ICC)

Cultured cells were fixed for 30 minutes in 4% paraformaldehyde in phosphate buffer saline (PBS) and cover slips were washed in PBS, treated with blocking buffer (PBS supplemented with 0.1% BSA, 0.1% Tween 20, and 0.1% NaNO_3_), and incubated with the respective antibodies as described [18, 26]. They were then rinsed in PBS and incubated with goat anti-rabbit IgG Alexa Fluor 488 or goat anti-mouse IgG Alexa Fluor 546 (monoclonal antibodies) (1 : 1000) for 1 h at room temperature (RT). In some experiments, samples were incubated with propidium iodide (PI) (0.05 mg/mL). Cover slips were finally washed thoroughly and visualized by confocal microscopy (FV1000; Olympus, Tokyo, Japan).

### 2.6. RNA Isolation and RT-PCR

Total RNA from RGC or HC primary cultures was extracted following the method of Chomczynski and Sacchi using the TRIzol kit for RNA isolation (Invitrogen) as previously described [17]. RNA integrity was checked in 1.5% agarose gel and quantified by UV spectrophotometry (Gene Quant spectrophotometer, Amersham Biosciences). Finally, 1-2 *μ*g of total RNA was treated with DNAse (Promega) to eliminate contaminating genomic DNA. cDNA was synthesized with M-MLV (Promega) using oligo (dT).

The oligonucleotide sequences used for RT-PCR from the* Gallus gallus* sequences were as follows [27]: 

*Bmal1*

Forward:  5′ TGAGGAGTCGCTGGTTCAGTTTCA 3′

Reverse: 5′ ACGCTGTCCATGCTATGTGGAGAA 3′

*GAPDH*

Forward: 5′ AGG CGA GAT GGT GAA AGT CG 3′

Reverse: 5′ TCT GCC CAT TTG ATG TTG CT 3′

*Cry 1*

Forward:  5′ AGAGAGTGTCCAGAAGGCTGCAAA 3′

Reverse:  5′ ACTGTTGCAAGAAGACCCAGTCCT 3′

*Cry 2*

Forward: 5′ CCA AGT GCA TCA TTG GAG TGG 3′

Reverse: 5′ CTT CAG TGC ACA GCT CTT CTG CTC 3′

*AA-NAT*

Forward: 5′ ACAGGCACCTTTACAGCACGAGA 3′

Reverse:  5′ CTGCTTCACGACAAACCAAGGCAT 3′

*Clock*

Forward:  5′ ACGGTCAAGGACTGCAGATGTTCT 3′

Reverse:  5′ CTGCAAAGGCTGTTGCTGGATCAT 3′

*Per 2*

Forward:  5′ TGGTCACCGTCAGACACTTCACAA 3′

Reverse: 5′ TTTCCCGAGTCTGGCAGCTGATTA 3′

*NPAS2*

Forward:  5′ CCAGGGCAAATTGCATCTCCACAA 3′

Reverse:  5′ AGGATGTGGGCATCATAGGCTGAA 3′

### 2.7. Polymerase Chain Reaction (PCR)

PCR reactions were carried out according to [17] with an initial denaturation step of 3 min at 95°C followed by 36 cycles of denaturation at 95°C for 15 sec, annealing at 60°C for 30 sec, extension at 72°C for 30 sec, and a final 5 min elongation step at 72°C. Amplification products were separated by agarose gel electrophoresis and visualized by ethidium bromide staining.

### 2.8. Inositol Phosphates (IPs) Assessment

RGC cultures were metabolically labeled with 2 *μ*Ci·mL^−1^ of myo-[2-^3^H(N)] inositol (PerkinElmer Life and Analytical Sciences) during 48 h. The cells were then stimulated with cool white fluorescence light (1200 lux) during different times according to conditions used to depolarize ipRGCs in mammals [28], in the presence of 10 mM LiCl. The lipids were recovered by TCA extraction methods [29–31] and the inositol phosphates (IPs) were recovered from the protein/membrane pellets [32]. IPs were then separated by Dowex AG1-X8 columns and eluted with increasing concentrations of ammonium formate and formic acid as described [29, 30]. The radiolabeled IP content was determined in a scintillation counter.

### 2.9. Statistics

Statistical analyses involved a one-way analysis of variance (ANOVA) with Duncan post hoc tests or Student's *t*-tests, when appropriate (significance at *P* < 0.05).

## 3. Results and Discussion

**Figure 1:**
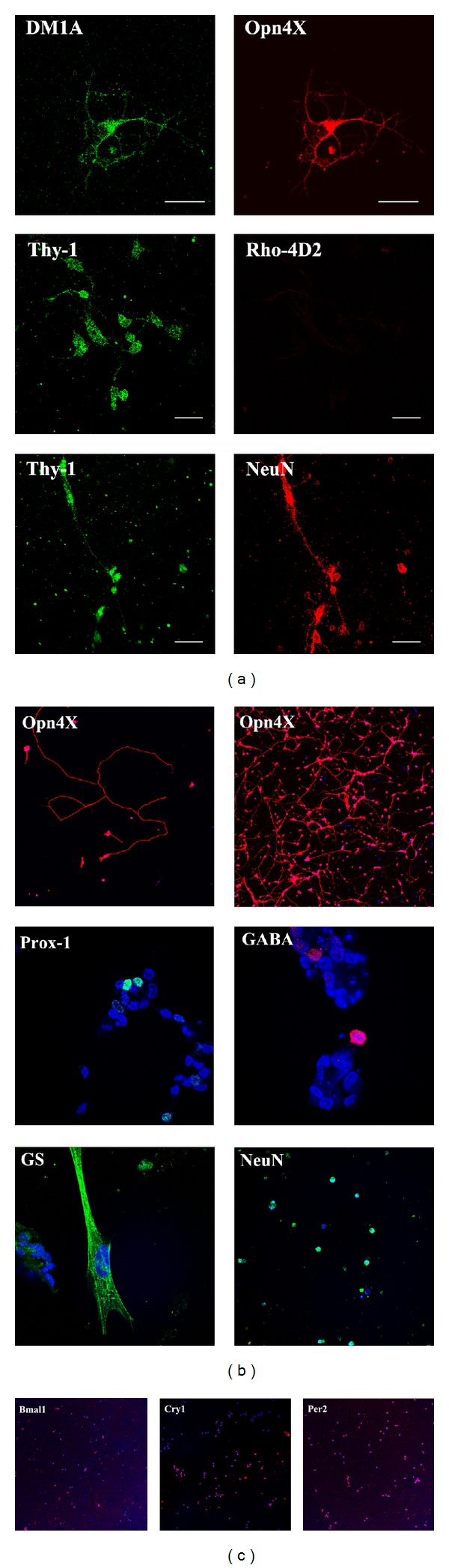
Immunocytochemistry for immunopurified RGC primary cultures at embryonic day 8 (E8) by Thy-1 (a), Opn4x (b) antibody purifications, and clock genes of RGC* Opn4x* cultures. (a) Primary cultures of embryonic RGCs purified by Thy-1 antibody immunopanning and maintained for 48–72 h were immunolabeled for DM1A, Opn4x, Thy-1, Rhod-4D2, and NeuN. (b) Immunocytochemistry for Opn4x- Prox-1, GABA, glutamine synthetase (GS), and NeuN, with DAPI staining in purified RGC cultures at E8 by Opn4x- antibody immunopurification. Primary cultures were visualized by confocal microscopy with specific primary antibodies as described in Section 2. Scale bar = 20 *μ*m. (c) Primary cultures of RGC's immunopurified by Opn4x-antibody immunolabeled with anti-Bmal1, Cry1, and Per2.4.

**Figure 2:**
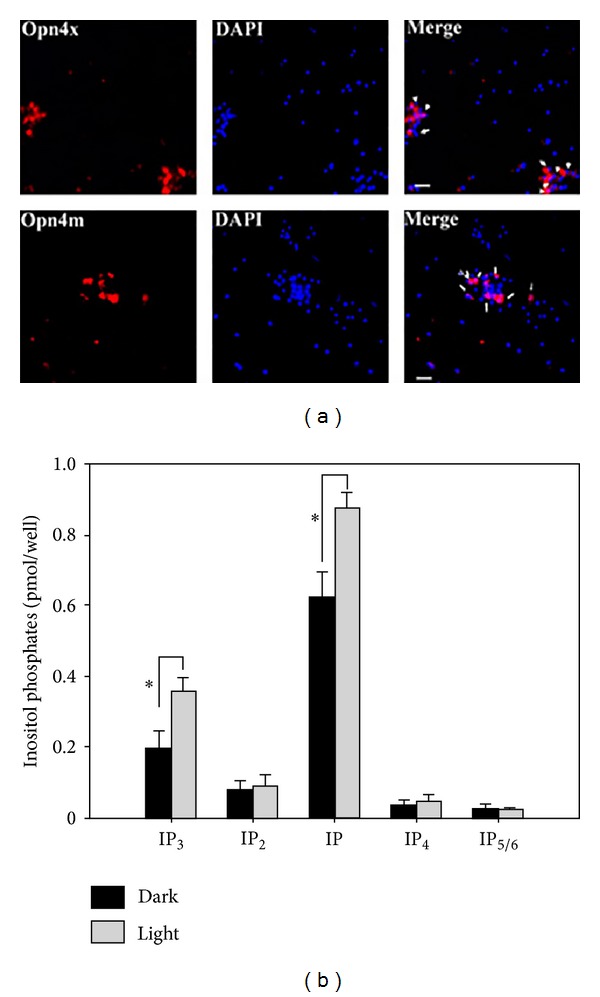
Melanopsin expression and light responses in RGC cultures. (a) Primary cultures of embryonic RGCs maintained for 48–72 h were immunolabeled for Opn4x or Opn4m with specific antibodies and DAPI (nuclei staining). The white arrows identify clusters of Opn4 (+) cells present in RGC cultures observed at 40X. (b) Content of inositol phosphates (IPs) in primary cultures of RGCs maintained in the dark (black squares) or stimulated by light (gray squares). Cultures previously incubated with myo-^3^H inositol for 48 h were light-stimulated during 90 sec to investigate the generation of different IP derivatives directly in RGCs. IP production was evaluated after bright light stimuli and control cells were maintained in the dark as described in the text. Significant increases in radiolabeled IP_3_ and IP were seen in cultures exposed to bright white light as compared with dark controls (*P* < 0.04). On the contrary, no significant light-dark differences were found in the content of other IPs determined such as IP_2_, IP_4_, and IP_5/6_. See text for further details.

We first examined the expression of Opn4 and a number of retinal cell type markers in primary cultures of RGCs obtained either by Thy-1-(Figure 1(a)) or* Opn4x*-antibody immunopurifications (Figure 1(b)) at embryonic day 8 (E8). Primary cell cultures were obtained by Thy-1 immunopanning, expressed Opn4x (Figures 1(a) and 2) and Opn4m (Figure 2), and exhibited a positive immunoreactivity for the RGC markers NeuN and Thy-1 in most cells but not for rhodopsin, a typical PRC marker (Figure 1(a)). When the primary RGC cultures were prepared by Opn4x immunopurification at E8, most cells expressed Opn4x and NeuN while only a very few (<10%) exhibited positive immunostaining for HC markers such as Prox-1 or GABA or for the glial cell marker GS (Figure 1(b)). In addition, the immunoreactivity associated with Opn4x clearly labeled all RGC neurite processes, even the longest ones (see stained individual cells in upper panel of Figures 1(a) and 1(b)). Moreover, Opn4x (+) RGC cultures were also found to express different clock proteins such as the Bmal1, Cry1, and Per2 (Figure 1(c)). To further characterize the expression of both Opn4 proteins in RGC primary cultures obtained by Thy-1 antibody immunopurification at E8 and their photic responsiveness, we carried out a series of new experiments shown in Figure 2. RGC cultures displayed a positive immunoreactivity associated with Opn4x or Opn4m in approximately 11% and 22% of total cells, respectively, in agreement with observations previously reported [18]. In addition, we further investigated the intrinsic light responsiveness of the primary RGC cultures shown in Figure 2 by assessing the formation of different radiolabeled inositol phosphates (IPs) (IP, IP_2_, IP_3_, IP_4_, and IP_5/6_) after light stimulation and comparing it with cultures kept in the dark. A very rapid generation of radiolabeled IPs occurred in the light by activation of the phospholipase C (PLC) which was previously shown to be involved in the RGC phototransduction cascade [17, 18]. In this connection, RGC cultures previously incubated with myo-^3^H inositol were light-stimulated during 90 sec or maintained in the dark (controls) in the presence of LiCl (20 mM), a well-known inositol monophosphate phosphatase inhibitor, in order to assess levels of different IPs under both light conditions. We found a significant increase in labeled IP_3_ and IP in cultures exposed to bright white light as compared with controls kept in the dark (Figure 2(b)); this increase represents a 70% and 40% rise in IP content, respectively, compared to basal levels (*P* < 0.04). By contrast, no significant light-dark differences were found in the content of other IPs determined such as IP_2_, IP_4_, and IP_5/6_, likely reflecting the very fast metabolism of these particular IP derivatives.

**Figure 3:**
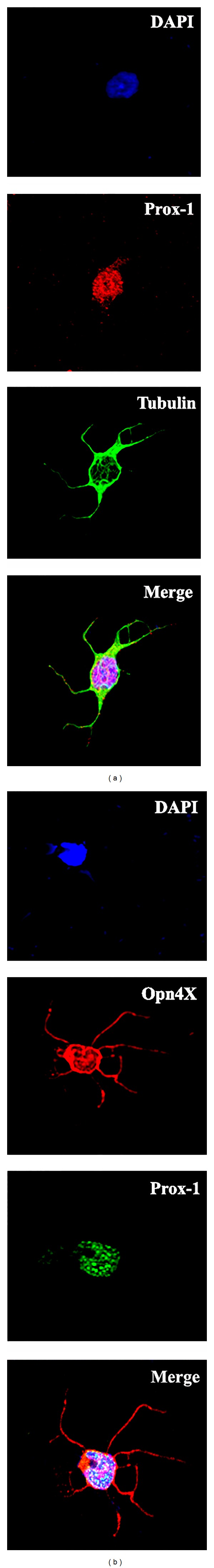
Immunocytochemistry for Prox-1, Opn4x, and *α*-Tubulin proteins staining and merge in HC cultures at E15. Individual cells from primary cultures of HCs were obtained by the 2.5% phase of a bovine albumin serum (BSA) gradientpurification, maintained for 48–72 h, immunolabeled for Opn4x-like protein ((b) red), *α*-Tubulin (DM1A) ((a) green), Prox-1 ((a) red; (b) green), and nuclei staining by DAPI (blue), and then visualized by confocal microscopy as described in Section 2.

Opn4x-like protein was strongly visualized in the OPL of the chicken retina at a later developmental/postnatal stage. Moreover, Opn4x was strongly expressed in Prox-1(+) cells localized in the inner nuclear layer of the avian retina [21]. More recently, we described a protocol using a BSA gradient to obtain primary cultures highly enriched in HCs at embryonic day 15 [26]. By means of this purification procedure, in this paper, we have further characterized the 2.5% phase containing HCs. As shown in Figure 3, primary cultures highly enriched in HCs exhibiting positive immunoreactivity for Prox-1, a typical nuclear HC marker, colocalized with the fluorescence for Opn4x. Interestingly,* Opn4x* labeling was concentrated in both soma and neurites of cultured cells; these observations are in clear agreement with the immunostaining observed in our previous observations [21] at E15 and posthatch days. In addition, this figure also shows colocalization of Tubulin with Prox-1 to clearly denote the typical HC morphology.

**Figure 4:**
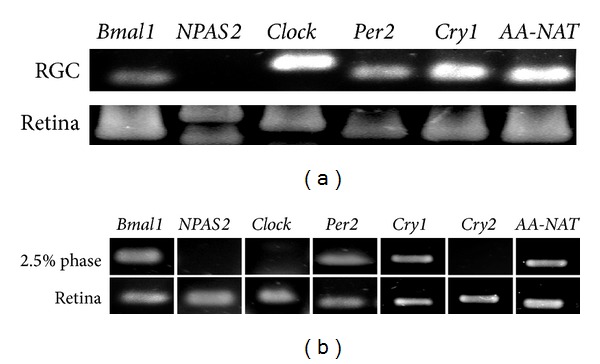
Analysis of mRNA expression in primary cultures of chicken RGCs at E8 (a) and of HCs at E15 (b) and in the whole postnatal chicken retina. (a) Chicken embryonic retinas were dissected out at E8 and RGCs were purified and cultured. Expression of clock genes cryptochrome 1 (*Cry 1*),* Clock*,* Bmal1*,* NPAS2*, and* Per 2* and clock-outputs: the melatonin synthesizing enzyme, arylalkylamine* N*-acetyltransferase (AA-NAT) mRNA was assessed by the reverse transcription- (RT-) polymerase chain reaction (PCR).* Cry 1*,* Per2 Clock*,* Bmal1*, and* AA-NAT* PCR products were found in RGC cultures whereas no detectable amplification was found for the* NPAS2* transcript. (b) Chicken embryonic retinas were dissected out at E15, and HCs were purified and cultured. mRNA expression for clock genes cryptochromes 1 (*Cry 1*) and 2 (*Cry 2*),* Bmal 1*, and* Clock* and for the clock-outputs AA-NAT was assessed by RT-PCR from HCs at E15 (phase 2.5%) and samples from the whole postnatal retina (positive control). Positive amplification was found for the mRNAs of* Cry 1*,* Per2*,* Bmal1*, and* AANAT *whereas* Cry2*,* NPAS2*, and* Clock* amplifications products were not found in HC cultures.

We then investigated the expression of clock genes and circadian markers in primary cultures of RGCs and HCs and compared it with expression of markers in the whole mature retina. RGC cultures immunopurified by Opn4x-antibody purification at a very early embryonic day (E8) express the transcripts for positive elements of the molecular clock such as* Bmal1* and* Clock* but not for* NPAS2*, which are strongly present in the whole retina (Figure 4); Opn4 (+) RGCs also express the negative elements of the molecular clock such as* Per2* and* Cry1* as well as the mRNA for* AA-NAT*, the key enzyme for melatonin biosynthesis (Figure 4).

In addition, highly enriched primary cultures of HCs, prepared with the 2.5% phase of the BSA gradient, clearly expressed the clock genes* Bmal1*,* Cry1*, and* Per2* as well as* AA-NAT* (Figure 4). By contrast, the transcripts for* NPAS2* and* Clock* were not detected in the cultures, although they were clearly visualized in the positive controls (the whole mature chicken retina). These findings demonstrate the presence of some clock and clock-related genes in the cultures, allowing us to infer that Opn4x and components of the molecular clock are present in the same cell population. Although it has been previously reported that embryonic retinal cell cultures in the chicken express clock genes [7], this is the first time that the presence of clock genes and AA-NAT together has been shown in primary cultures of both Opn4x (+) RGCs and isolated HCs in the developing retina when PRCs are not yet functional [33]. Based on these observations we may infer that inner retinal cells (RGCs and HCs) contain components of the molecular and genetic machinery for endogenous rhythm generation. Moreover, highly enriched preparations of inner retinal cells containing HCs, among other cells, from mature lyophilized retinas, display detectable levels of AA-NAT activity (data not shown) with values closely related to those found in RGCs [8]. In addition, it has been reported that AA-NAT levels can vary along with variation of the intracellular Ca^+2^ content [34]. In this respect,* Opn4x* expressed in chicken HCs, if functionally photoactive, could be implicated in the putative mechanism of AA-NAT variation likely triggered by light stimulation. Nevertheless, further studies will be required to demonstrate the intrinsic photoreceptive capacity of these cells in the chicken retina.

Remarkably, visual photoreceptor cells (cones and rods) and ipRGCs in the vertebrate retina together with oscillators located in the pineal gland of nonmammalian vertebrates are all photoreceptive and capable of producing melatonin in a circadian fashion [1]. Although HCs express the photopigment* Opn4x* and exhibit detectable levels of clock genes and* AA-NAT* mRNAs and appreciable levels of enzyme activity were found in inner retinal preparations of postnatal retinas (data not shown), future research will address the potential capacity of HCs to synthesize acetylserotonin and/or more complex methoxyindoles as well as their potential intrinsic responsiveness to light.

## 4. Conclusions

In this work, we have further characterized primary cultures of two different populations of inner retinal cells (RGCs and HCs) at early developmental stages (E8–E15) that express the nonvisual photopigment Opn4x. Opn4 has been shown to confer intrinsic photosensitivity on nonretinal cells [35, 36] and to be responsible for light detection, regulating a number of nonvisual activities (synchronization of biological rhythms, suppression of pineal melatonin, sleep, etc) in mammals [37] and nonmammalian vertebrates [1]. Overall, the expression of this novel opsin as well as that for different circadian markers such as the clock genes* Bmal1*,* Per*, and* Cry* and the key melatonin synthesizing enzyme, AA-NAT, appears very early in development in RGCs and HCs, even before any sign of formal vision takes place. Inner retinal cells may therefore acquire the capacity to both sense ambient light conditions and measure time very early in development, which may help to improve the adjustment of retinal clock physiology. In this context, melatonin can act as the nocturnal circadian marker in the outer retina and together with dopamine as a diurnal signal in the inner retina regulating the function of local circuits [1]. Our observations clearly suggest that nonvisual Opn4 photoreceptors and endogenous clocks may converge in these inner retinal cell populations to further support the circadian timing system and to improve the temporal regulation of physiology.
